# Modulation of Coenzyme Q_10_ content and oxidative status in human dermal fibroblasts using HMG-CoA reductase inhibitor over a broad range of concentrations. From mitohormesis to mitochondrial dysfunction and accelerated aging

**DOI:** 10.18632/aging.101926

**Published:** 2019-05-10

**Authors:** Fabio Marcheggiani, Ilenia Cirilli, Patrick Orlando, Sonia Silvestri, Alexandra Vogelsang, Anja Knott, Thomas Blatt, Julia M. Weise, Luca Tiano

**Affiliations:** 1 Department of Life and Environmental Sciences, Polytechnic University of Marche, Ancona, Italy; 2 Department of Clinical and Dental Sciences, Polytechnic University of Marche, Ancona, Italy; 3 Research and Development, Beiersdorf AG, Hamburg, Germany

**Keywords:** Coenzyme Q_10_, aging, skin, mitochondria, oxidative stress, senescence

## Abstract

Coenzyme Q_10_ (CoQ_10_) is an endogenous lipophilic quinone, ubiquitous in biological membranes and endowed with antioxidant and bioenergetic properties, both crucial to the aging process. In fact, coenzyme Q_10_ synthesis is known to decrease with age in different tissues including skin. Moreover, synthesis can be inhibited by 3-hydroxy-3-methyl-glutaryl-coenzyme A (HMG-CoA) reductase inhibitors such as statins, that are widely used hypocholesterolemic drugs. They target a key enzymatic step along the mevalonate pathway, involved in the synthesis of both cholesterol and isoprenylated compounds including CoQ_10_.

In the present study, we show that pharmacological CoQ_10_ deprivation at concentrations of statins > 10000 nM triggers intracellular oxidative stress, mitochondrial dysfunction and generates cell death in human dermal fibroblasts (HDF). On the contrary, at lower statin concentrations, cells and mainly mitochondria, are able to partially adapt and prevent oxidative imbalance and overt mitochondrial toxicity. Importantly, our data demonstrate that CoQ_10_ decrease promotes mitochondrial permeability transition and bioenergetic dysfunction leading to premature aging of human dermal fibroblasts *in vitro*.

## Introduction

Coenzyme Q (CoQ_10_) is a lipophilic endogenous isoprenylated quinone widely distributed and conserved in living organisms. In human beings, the isoprenoid chain is composed of 10 units (CoQ_10_). Due to its ubiquitous presence in cellular membranes, it is also referred to as ubiquinone; in fact, it is present in lipoproteins as well as cellular and intracellular membranes.

CoQ_10_ has two main biological functions: it supports mitochondrial bioenergetics and in its reduced form, ubiquinol, it is a phenolic antioxidant that acts as an electron donor effective in inhibiting lipid peroxidation of biological membranes as well as exerting anti-apoptotic activity [1]. In fact, CoQ_10_ in its reduced form ubiquinol (CoQ_10_H_2_), is a potent antioxidant in the lipophilic environment, both by directly scavenging ROS and also by regenerating lipophilic and hydrophilic antioxidants such as tocopherol and vitamin C. Moreover, plasma membrane CoQ_10_H_2_ has been shown to exert an anti-apoptotic effect by counteracting the release of ceramide from sphingomyelin, hence regulating the extracellularly-induced ceramide-dependent programmed cell death pathway [2]. In this respect, Plasma Membrane Reductive Systems (PMRS) play a major role in activating CoQ_10_ through enzymatic two electron reduction catalyzed by dehydrogenase enzymes, mainly NADH-cytochrome b5 reductase and NAD(P)H: quinone reductase 1 (NQO1). This activity is influenced by the aging process and by pro-oxidant stimuli that may decrease the activity of membrane CoQ_10_ reductive systems [3]. In contrast, caloric restriction (CR) has been shown to up-regulate the plasma membrane redox system in brain cells and to suppress oxidative stress during aging. Moreover, recent studies have shown that over-expression of NQO1 in transgenic mice, was able to mimic the effects of CR by decreasing the levels of inflammation and neoplastic proliferation as well as enhancing bioenergetic activity [4].

CoQ_10_ within the mitochondrial membrane acts as an electron shuttle between complex I/II and complex III. The dynamics of the electron transfer, that was originally defined in a random collision model, has been recently updated by the identification of respiratory supercomplexes comprising complexes I and III [5,6]. In this updated view, part of mitochondrial CoQ_10_ is bound within supercomplexes where electron transfer would be mediated by tunneling or microdiffusion, with a clear kinetic advantage. CoQ_10_ content in mitochondria is in large excess compared to the prosthetic groups of respiratory enzymes [7] and the un-bound mitochondria CoQ_10_ pool, besides constituting a reservoir for bioenergetic requirements and an antioxidant for mitochondrial membrane lipids, is able to modulate bioenergetic processes and cell death pathways by binding to specific proteins, such as uncoupling proteins (UCPs) [8] and the permeability transition pore (PTP) [9].

Early reports identified physiological concentrations of CoQ_10_ in mitochondria in the range of the Km of the respiratory complex I and II. This led to the conclusion that slight variations in CoQ_10_ content may produce significant effects on the efficiency of the mitochondrial respiratory chain [10]. Recent evidence supporting the existence of respiratory chain complexes in supermolecular assemblies, called supercomplexes or respirasomes, partially challenges this view in light of optimization of electron transfer processes in comparison to the random collision hypothesis; nonetheless, it is also believed that CoQ_10_ content is in a dynamic equilibrium between the free CoQ_10_ pool and the supercomplex-bound form. As a consequence, mitochondrial CoQ_10_-pool decrease should also influence respiratory efficiency.

In fact, decrease in CoQ_10_ content is known to occur in conditions associated both with oxidative stress and mitochondrial dysfunction such as degenerative pathologies and in physiological conditions during the aging process with organ specific differences [11] both in animal models [12] and in humans [13,14]. Moreover, CoQ_10_ supplementation has been shown to be effective in reverting senescent phenotype features in *in vitro* models as well as in animal and human interventions studies. In this respect recent studies have shown that CoQ_10_ supplementation was able to counteract oxidative stress and inflammatory responses in senescent endothelial cells as well as decrease the expression of SASPs (Senescence Associated Secretory Phenotype) genes [15,16]. Co-treatment of CoQ_10_ and physical exercise in senescence accelerated mice (SAMP8) was efficient in slowing down degenerative processes in skeletal muscle cells by protecting mitochondria structure and function and inducing mitochondrial biogenesis [17]. Furthermore, stress-induced accelerated senescence in CoQ_10_ supplemented mice was able to decrease senescence markers, oxidative stress and fibrotic tissue remodeling in cardiac tissue [18,19].

Both antioxidant and bioenergetic roles of CoQ_10_ are therefore deeply related with the aging process. In fact, mitochondria are a critical site representing both a source and a target of reactive oxygen species (ROS). During cellular senescence, increased ROS release by mitochondria might trigger a vicious cycle involving accumulation of oxidative damage to mitochondrial membranes and respiratory complexes leading to enhanced ROS production [20-21-22]. This condition is associated with dynamic rearrangements of mitochondria, that in virtue of their elevated plasticity, are able to modulate their morphology and functionality in response to stress [23]. In particular, low levels of stress are able to activate signaling pathways through the activation of redox-sensitive transcription factors such as NRF2 that is involved in the transcription of detoxifying and antioxidant enzymes [24]. Chronic levels of stress however, result in extensive fragmentation of mitochondria, decrease in their copy number and lowered respiratory capacity [25,26].

Ultimately, mitochondria accumulating excessive damage are able to trigger apoptotic cell death processes, further stressing the pivotal role of these organelles in cellular life and death. In relation to these aspects, both bioenergetic and antioxidant functions of CoQ_10_ are relevant to maintain mitochondrial functionality and to prevent age-associated damage.

The human skin, due to its function as interface with external environment, is more exposed than other tissues to damaging pro-oxidant stimuli, such as high oxygen tension, ultraviolet radiation, and pollution, that enhance cellular oxidative stress and accelerate senescence processes. Photoaging of the skin and exposure to tobacco smoke represent classical examples of this process, promoting altered barrier function of the tissue, susceptibility to inflammatory processes and skin aging features such as mottled hyperpigmentation or dyspigmentation, loss of skin’s firmness and elasticity, and wrinkle development [27]. Among the skin’s antioxidant protection system, CoQ_10_ has a primary function in protecting lipid components from oxidation. Notably, CoQ_10_ content in the epidermis, the outermost layer of the skin, is up to ten times higher compared to the adjacent dermis [28]. The aging process is known to affect skin’s CoQ_10_ content similarly to other tissues [29] and strategies aiming to delay its deprivation in skin have been proposed as anti-aging approaches [30]. At the mitochondrial level, a CoQ_10_ decrease in dermal fibroblasts has been shown to be associated with a significant inhibition of the respiratory chain as a result of decreased activity of complexes I/III, and II/III, leading to mitochondrial membrane depolarization and increased release of superoxide anions [31]. Exogenous CoQ_10_ supplementation significantly protects from mitochondrial dysfunction and oxidative damage *in vitro* in photo-exposed keratinocytes and dermal fibroblasts. Moreover, topical application in healthy human subjects revealed significant photoprotection and amelioration of the features of aged skin [29,32].

**Figure 1:**
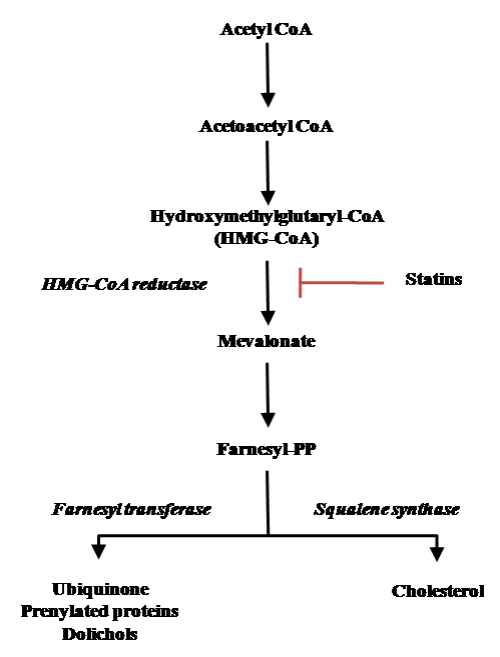
**The mevalonate pathway.**

CoQ_10_ cellular content can also be influenced by drugs interfering with its biosynthesis. Endogenous biosynthesis of CoQ_10_ is linked to the mevalonate pathway (Fig. 1) sharing precursors with cholesterol synthesis. Along this pathway, HMG-CoA reductase is a key enzyme regulating the synthesis of cholesterol as well as of CoQ_10_, dolichols and prenylated proteins. Dermal fibroblasts acquire exogenous cholesterol from adsorptive endocytosis of plasma low density lipoproteins (LDL) but are also able of endogenous biosynthesis through the mevalonate pathway [33]. Statins are selective inhibitors of HMG-CoA reductases widely used as hypocholesterolemic drugs in cardiovascular disease prevention. Although they are generally well tolerated at high doses, they may produce serious adverse effects such as rhabdomyolysis [20,34,35]. Several experimental evidence in cell culture and animal models link statin toxicity to mitochondrial impairment involving bioenergetics dysfunctions, enhanced oxidative stress, altered calcium homeostasis and programmed cell death activation [36-37-38]. While some recent data points out that simvastatin exerts a direct inhibitory effect on complex III [39] CoQ_10_ deprivation has clearly also a strong impact in producing these effects.

The aim of the present work is to establish a model of CoQ_10_ deprivation in human dermal fibroblasts in order to monitor how this affects intracellular oxidative status and mitochondrial functionality and to verify whether lowering CoQ_10_ levels is associated with the onset of senescence

## RESULTS

### Statin effect on cellular viability and CoQ_10_ biosynthesis

**Figure 2:**
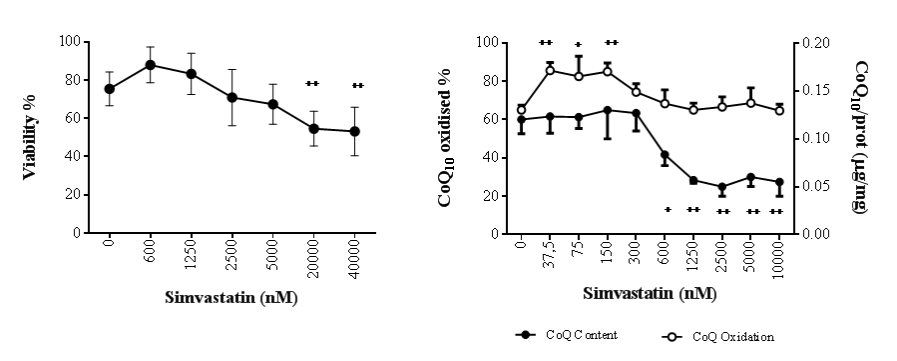
**Effect of simvastatin on cellular CoQ_10_ levels and cytotoxicity.** Viability (**A**) and CoQ_10_ content and oxidative status (**B**) of human dermal fibroblasts with different doses of simvastatin for 72 h. Data (n=9 A; n=4 B) are reported as mean and standard error of % live cells (A) and coenzyme Q_10_/total protein (μg/mg) (B). Significance difference vs 0 nM ^+^ p<0.05, ^++^ p<0.01, ^+++^ p<0.001, ^++++^ p<0.0001.

Statin exposure of fibroblasts for 72 h did not influence cell viability at concentrations lower than 20000 nM, while in the presence of 20000 and 40000 nM a significant drop in viability by ~25% was observed (^++^p<0.01) (Fig. 2A). Co-supplementation of statin 20000 nM with Coenzyme Q_10_ 10µg/ml was able to significantly counteract statin cytotoxicity to a lower extent in presence of the oxidized form (ubiquinone; ^+^p<0.05) and to a higher level of significance in presence of the reduced form (ubiquinol; ^++++^ p<0.0001) (Fig. S1). Simvastatin influences CoQ_10_ content and oxidative status in a dose-dependent manner (Fig. 2B): at concentrations lower than 300 nM, total CoQ_10_ content was not affected whereas a significant increase in its oxidation status was observed (+22% 37.5 nM, +17.5% 75 nM, +24% 150 nM; ^+^p<0.05, ^++^p<0.01). At increasing statin concentrations, the oxidative status of CoQ_10_ returned to comparable values of untreated control cells with a level of oxidation around 67%. In parallel, a significant decrease in CoQ_10_ content was observed starting from 600 nM simvastatin (-29%; ^+^p<0.05) reaching a plateau at 1250 nM and higher values (-44%; ^++^p<0.01).

### Statin influences mitochondrial and cellular reactive oxygen species levels in HDF and senescence markers

**Figure 3:**
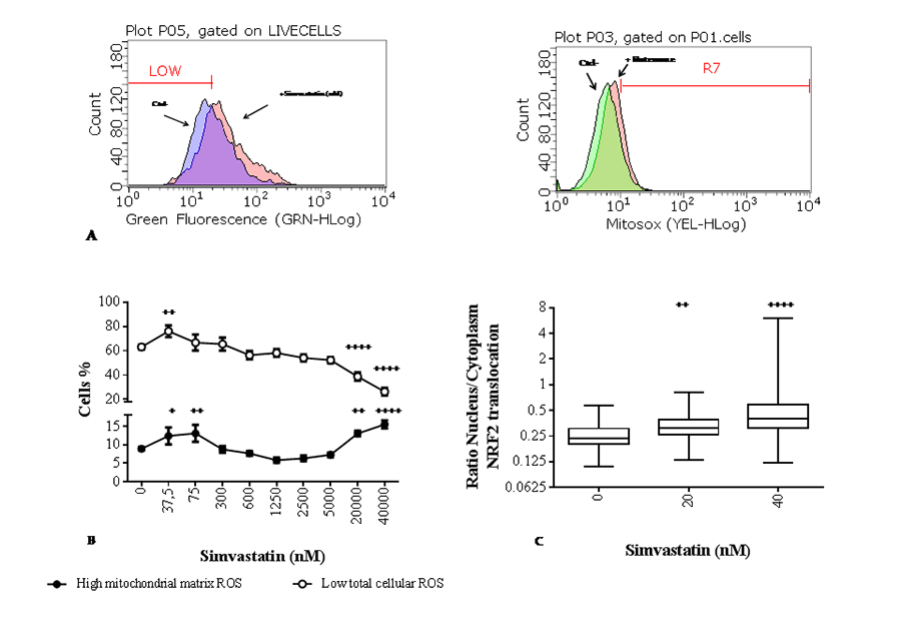
**Effect of simvastatin on reactive oxygen species (ROS) levels and nuclear factor (erythroid-derived 2)-like 2 (NRF2) translocation.** (**A**) Flow cytometric distribution of cells according to their cellular (DCF) and mitochondrial matrix (Mitosox) ROS content in a reference experiment to define gates relative to low and high ROS respectively. (**B**) Percentage of MitoSOX positive human dermal fibroblasts with elevated mitochondrial superoxide anion (●) and DCF negative cells with low cellular reactive oxygen species (○) after exposure to different concentrations of simvastatin for 72 h. Data (n=9) are reported as mean % of cells and standard error. (**C**) Nuclear translocation of NRF2 in simvastatin treated samples for 72 h (20 and 40 nM) and unexposed control. Data are expressed as ratio of NRF2-FITC associated green fluorescence in the nucleus and cytoplasm. Data are reported as box-plot with 50% of the population reported in the box; horizontal lines indicate min, median, and max values. Significance difference vs 0 nM ^+^ p<0.05, ^++^ p<0.01, ^+++^ p<0.001, ^++++^ p<0.0001.

Statin incubation for 72 h at concentrations lower than 300 nM increases slightly but significantly mitochondrial superoxide anion production; this increase was paralleled by a contrasting improvement in cellular oxidative status represented by a significant increase in the percentage of cells with LOW cellular ROS levels in the presence of 37 nM simvastatin (+15%, ^++^p<0.01) (Fig. 3B).

Increasing the concentration of statin over 300 nM, results in a progressive increase in cellular ROS with a population characterized by LOW ROS content comparable with the unexposed control up to 5000 nM. However, this population is significantly lowered in cells exposed to simvastatin 20000 nM (-23%, ^++++^p<0.0001) and 40000 nM (-32%, ^++++^p<0.0001) (Fig. 3B). Interestingly, at these higher concentrations mitochondrial superoxide production is also significantly higher than in untreated control cells; whereas in the intermediate range of exposure between 300 nM and 5000 nM mitochondrial superoxide release remains unchanged compared to untreated cells (Fig. 3B). In the low concentration range, significant modulation of cellular ROS content and mitochondrial O_2_^•−^, production is associated with significant nuclear translocation of NRF2 (Fig. 3C).

**Figure 4:**
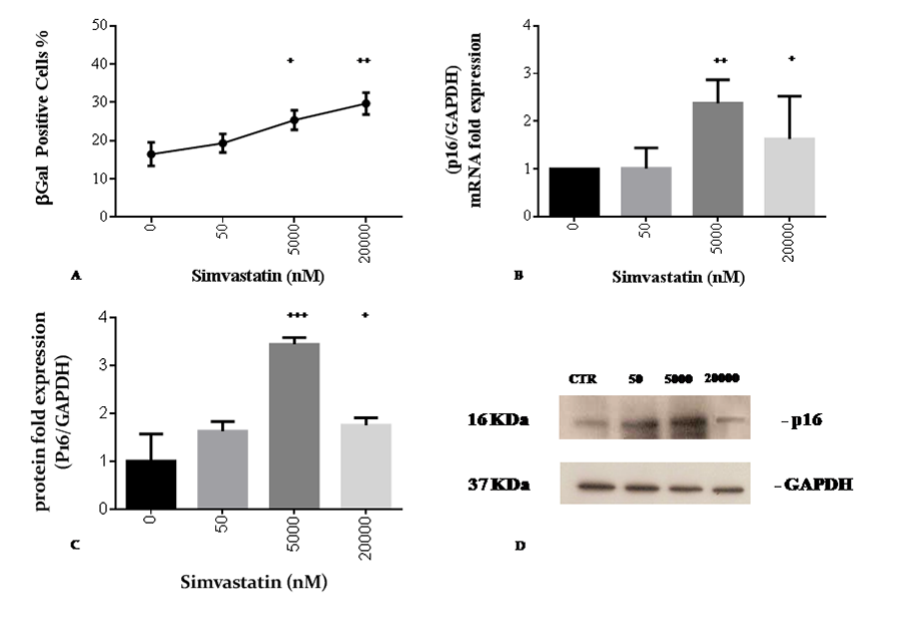
**Simvastatin promotes senescence markers of cellular senescence in human dermal fibroblasts incubated with different doses of simvastatin for 72 h.** Beta-galactosidase positive cells (**A**). p16 mRNA expression (**B**); western blot analysis of p16 (**C, D**). Significance difference vs 0 nM ^+^ p<0.05, ^++^ p<0.01, ^+++^ p<0.001, ^++++^ p<0.0001.

Altered cellular oxidative status is able to promote stress induced premature senescence as documented by a dose-dependent increase in ß-gal positivity, with significant differences compared to control cells at concentrations of 5000 and 20000 nM simvastatin (Fig. 4A). These data are confirmed at molecular level by a significant increase in the expression of p16 mRNA (Fig. 4B) and protein at 5000 nM and 20000 nM statin (Fig. 4C, D).

### Statin influences mitochondrial permeability transition pore, mitochondrial biogenesis and respiratory profile

A closer analysis of mitochondrial parameters shows that in relation to CoQ_10_ deprivation, mitochondria undergo relevant changes in their functionality and biogenesis.

**Figure 5:**
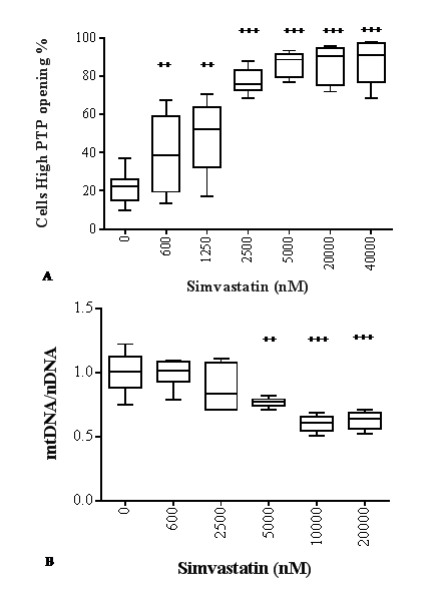
**Effect of simvastatin on mitochondrial toxicity.** Permeability transition pore opening (**A**) and mtDNA copy number (**B**) in human dermal fibroblasts in presence of different concentrations of simvastatin for 72 h. Data (n=12 A; n=6 B) are reported as box-plot with 50% of the population reported in the box; horizontal lines indicate min, median, and max values. Significance difference vs 0 nM ^+^ p<0.05, ^++^ p<0.01, ^+++^ p<0.001, ^++++^ p<0.0001.

In particular, in the presence of 600 nM simvastatin a significant mitochondrial transition pore opening is observed. This stress reaction is characterized by a wide distribution of cells indicating a heterogeneous behavior. Notably, mPTP opening increases dose-dependently up to 2500 nM (Fig. 5A) in parallel with the dynamics of CoQ_10_ depletion (Fig. 2B). Concentrations of statin > 5000 nM are associated with a consistent and significant decrease in cell viability (Fig. 2A), and the extent of permeability transition stabilizes around maximal levels (Fig. 5A) in correlation with CoQ_10_ decrease (Fig. 2B). Following incubation with 5000 nM statin, a significant decrease (-23%, ^+++^p<0.001) in mtDNA/nDNA ratio is also observed indicating increased mtDNA damage as a consequence of increased mitochondrial damage and dysfunction. This could suggest a decrease in mitochondrial copy number that is exacerbated in the presence of higher simvastatin concentrations (10000 nM and 20000 nM -39%, ^+++^p<0.001) (Fig. 5B).

**Figure 6:**
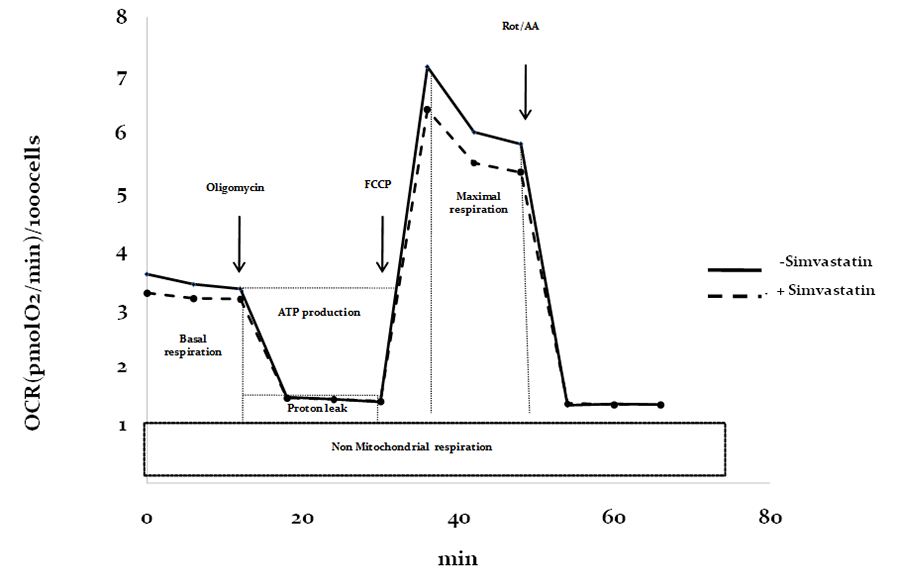
**Respiratory profile in control and statin-treated fibroblasts.** Reference of respiratory profile and outline of mitostress kit (Agilent) parameter calculation using specific mitochondrial respiratory inhibitors (oligomycin, FCCP, Rot/AA).

High-throughput respirometry analysis of HDF (calculated as pmol O_2_/min/1000 cells) in the intermediate-high range of simvastatin exposure (600-10000 nM) is reported in Fig.6.

**Figure 7:**
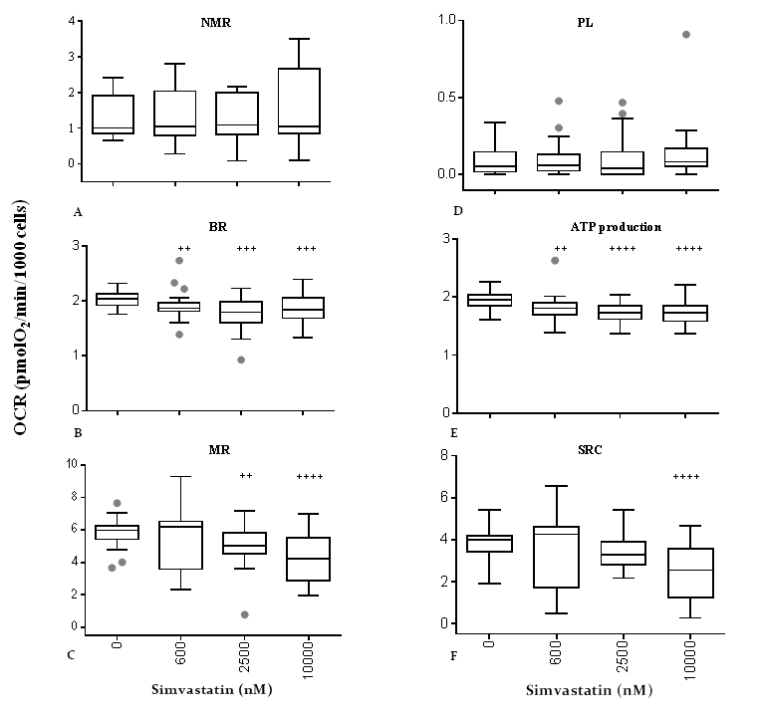
**Effect of simvastatin on mitochondrial respiratory indexes.** Respiratory profile of human dermal fibroblasts incubated with different concentrations of simvastatin for 72 h. Non-mitochondrial respiration (**A**), basal respiration (**B**), maximal respiration (**C**), proton leakage (**D**), ATP production (**E**), spare respiratory capacity (**F**). Data (n=48) are reported as box-plot values of oxygen consumption rate. Significance difference vs 0 nM ^+^ p<0.05, ^++^ p<0.01, ^+++^ p<0.001, ^++++^ p<0.0001.

The data show that simvastatin is able to decrease basal respiration (Fig. 7B) and consequently ATP production (Fig. 7E) already at 600 nM (-8.7% for basal respiration and -7.7% for ATP production, ^++^p<0.01) and to a higher extent at 2500 nM (-12.3% for basal respiration, ^+++^p<0.001, -11.5% for ATP production, ^++++^p<0.0001) and 10000 nM (-9.8% for basal respiration, ^+++^p<0.001, -11.5% for ATP production, ^++++^p<0.0001). However, maximal mitochondrial respiration (Fig. 7C) is comparable to untreated control cells in the presence of 600 nM simvastatin and dose-dependently decreases following incubation with simvastatin 2500 nM (-16%, ^++^p<0.01) and 10000 nM (-29%, ^++++^p<0.0001).

Overall, spare respiratory capacity (Fig. 7F), that is calculated from the difference between the rate of maximal respiratory capacity and basal respiration and which represents the ability of the cell to deal with an energetic crisis, was affected only in presence of 10000 nM simvastatin and not at lower concentrations.

Non-mitochondrial respiration and proton leakage (Fig. 7A, D) were not affected by any of the tested simvastatin concentrations confirming the integrity of the electron transport chain in the proposed experimental conditions up to 10000 nM.

## DISCUSSION

Simvastatin was used in our experimental model in virtue of its high bioavailability compared to other types of statins [40,41] and its use was mainly aimed at inhibiting CoQ_10_ synthesis and to evaluate the effect of its deprivation on cellular oxidative status, mitochondrial dysfunction and consequently development of senescence markers. In particular, a wide range of concentrations was used that were defined as low (<600 nM) intermediate (< 5000 nM) and high (> 10000 nM) by taking into account both statin efficacy in lowering CoQ_10_ content and its toxic effect. Interestingly, several pharmacodynamic human studies show that the usual posology of statin (40-80 mg/day) leads to plasma levels between 25 and 80 nM over 24 h after a single intake [42]. According to this data, on average, human plasma levels are well contained within our low experimental range, a condition that *in vitro* was not able to significantly inhibit CoQ_10_ synthesis but rather to affect its oxidative status.

This effect might be linked to the observed slight increase in mitochondrial superoxide anion production that is well tolerated by the cells. In fact, under this condition, cellular oxidative stress is low, or even decreased in comparison to untreated control, possibly as a result of adaptative mechanisms that could promote endogenous antioxidant defenses such as a hormesis. This hypothesis is supported by significant nuclear translocation of NRF2 that could be triggered by mild mitochondrial damage at low statin dosage. In fact, NRF2 nuclear translocation is influenced by electrophiles that are able to promote Keap-1 repressor ubiquitination [43]. Moreover, antioxidants such as tert-Butylhydroquinone (tBHQ) and natural bioactive compounds such as sulforaphane, are able to trigger this pathway by other pathways that might involve activation of cysteine residues [44].

Moreover, in a recent paper by Liu et al., it was shown that doses of statins compatible with the low dose range used in our experimental model, efficiently suppressed SASP in senescent cells *in vitro* without affecting the senescent growth arrest [45]. The authors suggest that these pleiotropic effects of statins are ascribed to lowering of protein prenylation inhibition and might have important implications in preventing the inflammatory microenvironment that promotes cancerous cell development. In our experimental setting in the low range of statin exposure, we did not observe any significant increase in stress-induced senescence markers, suggesting that minimal exposure to simvastatin might promote an adaptive response to support cellular oxidative defenses as long as physiologic cellular CoQ_10_ content is maintained. Accordingly, a key role of CoQ_10_ in triggering hormetic mitochondrial ROS signaling has been recently shown in insects and related to life span extension [46].

If on the one hand these evidences support safety of statins and provide molecular details behind the pleiotropic effect of statins, they should be considered carefully since acute exposure for 72h in our experimental model is not comparable to chronic exposure and relevant outcomes for human physiology should be verified *iv vivo*. In this respect, mitohormetic effects of simvastatins as HMG-CoA reductase inhibitors have been described in cardiac muscles from patients treated with statins as well as in cellular and animal murine models. It was shown that in cardiac tissues, statins triggered an increased expression of SOD 1, SOD 2 and PGC-1α supporting mitochondrial biogenesis and functionality while lowering ROS production through an adaptive mechanism. On the contrary, in skeletal muscles, statins produced an opposite effect leading to enhanced oxidative stress and decreased mitochondrial function and biogenesis, highlighting the fact that intracellular oxidative status is critical in steering cellular biochemical pathways toward adaptive response or cellular dysfunction [47].

Effective dosage required to produce CoQ_10_ deprivation started from concentrations that we defined as intermediate range concentrations that have been previously used in *in vitro* studies to evaluate statin-induced mitochondrial impairment in human skeletal myotubes [37]. Concentrations in the high range are comparable to the maximal concentrations used in similar *in vitro* studies [38]. In these conditions, after 3 days of exposure, simvastatin in our experimental model, produced an evident and dose dependent effect on cellular CoQ_10_ content and oxidative status with profound implications in mitochondrial functionality and cellular aging as summarized in Table 1.

**Table 1:** Effect of different simvastatin concentrations on cellular coenzyme Q10 and mitochondrial functionality.

	**Low range** **(<600 nM)**	**Intermediate range** **(600-5000 nM)**	**High range** **(>10000 nM)**
*Cellular CoQ__10__*	=	▼- ▼▼	▼▼
*CoQ__10__ oxidation*	▲	=	=
*Intracellular ROS levels*	(▼)	=	▲▲
*Superoxide Anion levels*	▲	=	▲
*Viability*	=	=	▼▼
*MPTP*	=	▲▲▲	▲▲▲
*mtDNA*	=	▼	▼▼
*Senescence marker*	=	▲	▲▲

= no effect, ▴ low / ▴▴moderate / ▴▴▴ strong increase, ▾ low / ▾▾ moderate / ▾▾▾ strong decrease

Differently, in the high range of exposure, endogenous CoQ_10_ synthesis is significantly affected with a decrease in endogenous biosynthesis of -44%. In our model, CoQ_10_ deprivation had important implications in terms of promoting mitochondrial derived oxidative stress that the cell is not able to counteract. As a consequence, in the high range of statin exposure, we observed a highly significant increase in mitochondrial superoxide anion production as well as an accumulation of cellular ROS. This condition is likely to be associated with the observed irreversible permeability transition pore opening in the decimated and dysfunctional mitochondrial population as suggested by the drastic decrease in mtDNA/nDNA ratio and the significant decrease in mitochondrial spare respiratory capacity. As a result programmed cell death programs are initiated [48]. Mitochondrial dysfunction and related oxidative stress have been shown to be associated in different experimental models with senescence induction [49,50]. Similarly, in our experimental model, high doses of simvastatin lead to significant decrease in viability and manifestation of a senescent phenotype in the residual cellular population. In this respect, markers of cellular senescence in terms of β-galactosidase, p16 mRNA and protein levels start to appear in the presence of lower levels of statin (5000 nM). In particular, p16 expression seems to be lower at 20000 nM compared to 5000 nM.

This apparent contradiction could be associated with a higher level of toxicity of statin treatment at high dosage which affects protein expression systems. Moreover, in light of a significant decrease in viability, a selection of a subpopulation of cells more resistant to senescence could not be ruled out.

Similarly, modulation of the mPTP and enhanced ROS formation accompanied by respiratory complex dysfunction observed in fibroblasts derived from patients with primary CoQ_10_ deficiency, were associated with lysosomal proliferation and activation of mitophagic processes, a specific subtype of autophagy aimed at selectively eliminating damaged mitochondria [51]. In the presence of mild oxidative stress, mitophagy is an efficient process in supporting mitochondrial turnover [52]. However, in enhanced oxidative stress conditions, such as those produced in our experimental model in the presence of high doses of simvastatin, this mechanism does not seem to be sufficient for removing dysfunctional mitochondria as suggested by the remarkable decrease in mtDNA copy number. This marker, in fact, is an additional index of damage to mitochondria and has been used to also support the decrease in mitochondrial mass.

Finally, exposure to the intermediate range of simvastatin leads to a complex biochemical scenario where cellular rearrangements seem to be efficient in actively counteracting statin induced CoQ_10_ deprivation and its oxidative consequences. In fact, while CoQ_10_ content is already significantly decreased at concentrations of simvastatin as low as 600 nM, mitochondrial functionality is only partially impaired since respiration proceeds at a lower rate. Notably, the spare respiratory capacity is not altered supporting the ability of the rearranged mitochondrial networks to deal with an energetic crisis.

Within the broad range of concentrations tested (600-5000 nM), cellular viability is not compromised but characteristic features of senescent cells are detectable such as increased numbers of senescence-associated ß-gal positive cells, dose-dependent decrease in mitochondrial content and mPTP opening. It is logical to assume that under these conditions, characterized by mild CoQ_10_ deprivation such as those experienced during the aging process [11,29], mitochondria are able to dynamically respond by rearranging the network and selectively eliminating dysfunctional units, and minimizing ROS release. In this sense, the proposed model suggests a statin-induced premature senescence model, highlighting the pivotal role of CoQ_10_ content in preventing cellular senescence independently of its antioxidant role. In fact, CoQ_10_ content and mitochondrial function are intimately linked and their levels are known to decrease with aging process. In this respect, Greco et al. [53] have shown in 51 dermal fibroblast cultures from individuals aged from 1 to 103 years, a clear age-related functional change in mitochondrial processes concerning the cellular oxidative phosphorylation capacity, like mitochondrial protein synthesis, respiration, and coupling of respiration to ATP synthesis with a significant decline in all these parameters, that becomes significant in cells from subjects around 40 years. In the present study, we show that we are able to produce several of these effects *in vitro* by decreasing CoQ_10_ content.

In support of the functional role of CoQ_10_ deprivation in promoting cellular senescence, we also showed that incubation with intermediate and high range of statin is able to significantly decrease CoQ_10_ content and promote cellular β-galactosidase activity in a dose dependent manner (Fig. 4A). In this respect, beside its acknowledged role as a marker of cellular senescence [54], the activity of this enzyme is also deeply connected to lysosomal function. Extra-mitochondrial coenzyme CoQ_10_ content is also relevant in the lysosomal compartment where it acts as electron and proton shuttle in the acidification process of vacuoles [55,56]. Therefore, CoQ_10_ depletion could also be associated with an increase in the pH of lysosomal lumen leading to enhanced synthesis of β-galactosidase as a compensatory mechanism implemented in order to maintain the efficacy of these organelles in removing non-degradable intracellular macromolecules [57].

In this context, extra-mitochondrial CoQ_10_ depletion could represent an additional trigger for cellular senescence induction, correlating with a modulation of lysosomal activity. This hypothesis deserves further investigation with regard to the critical role of these organelles in the senescence process [58,59]. Although simvastatin is an inhibitor of an early step in the mevalonate pathway, it may affect also other metabolites besides CoQ_10_ that might be responsible for the observed effects. Our results are in line with other experimental evidences that clearly link simvastatin-induced CoQ_10_ deprivation to mitochondrial dysfunction in *in vitro* studies. In particular, Tavintharan *et al.* showed in simvastatin treated HEPG2 cells, deprivation of CoQ_10_ levels associated with oxidative DNA damage, decreased ATP synthesis and promoting of cell death [60]. Similarly, Vaughan *et al.* in rhabdomyosarcoma cells, observed a dose dependent effect of statins in promoting mitochondrial dysfunction, decreasing ATP synthesis and overall mitochondrial content [61]. Notably, in both studies the detrimental effect of statins was prevented by CoQ_10_ supplementation.

## Conclusion

In conclusion, the present study strengthens and provides molecular evidences of the evident connection between CoQ_10_ content and cellular aging. Intriguingly, we also show that mitochondria and cellular biochemical systems are able to dynamically respond to CoQ_10_ deprivation by activating compensatory mechanisms that contain cellular oxidative stress in a wide range of concentrations of simvastatin tested, that are likely to represent a pre-senescence stage in dermal fibroblasts.

A limitation of the present study is represented by the potential effects of statins not directly related to CoQ_10_ deprivation. In fact, although CoQ_10_ synthesis was shown to be significantly affected by statins, HMG-CoA reductase constitutes an early step in the mevalonate pathway and its inhibition affects also other isoprenylated molecules involved in cellular biochemistry. The specific role of CoQ_10_ in mitochondrial dysfunction and senescence promotion will be verified in rescue experiments using CoQ_10_ supplementation to statin-deprived fibroblasts.

## MATERIALS AND METHODS

### Cell culture

Primary human dermal fibroblasts (HDF), were purchased from the Istituto Zooprofilattico Sperimentale, Brescia, Italy. Cells cultured at passage (10-20p) were used for the experiments and cultured in MEM supplemented with 10% fetal bovine serum (GENENCO, South America Origin), 1% penicillin (100 U/ml), 1% streptomycin (100 g/ml) and maintained in standard culture conditions at 37°C in a CO_2_ Heraeus BB15 incubator (Thermo Scientific) under a humidified atmosphere.

For cell culture maintenance, the medium was refreshed every 2–3 days and cells were passaged at over 80% confluence by trypsinization. For the experiments, cells were seeded in 24-well plates or in 6-well plates at an optimal density of 11x10^3^ cells/cm^2^.

### Simvastatin treatment

Simvastatin was purchased from Sigma-Aldrich diluted as a stock solution in DMSO at 25 mM and further diluted in cell culture medium in the range of 37.5 nM up to 40000 nM immediately preceding the incubation. Solvent control experiments were performed using DMSO only. Fibroblasts were incubated with different HMG-CoA reductase inhibitor concentrations for 72 h. In rescue experiments (supplementary material) cells where co-incubated with 10 µg/ml coenzyme Q_10_ either reduced (ubiquinol) or oxidized (ubiquinone). Coenzyme Q_10_ was solubilized in water using a mixture of glycerol and the emulsifying agent PEG 60-hydrogenated castor oil (Cremophor; BASF SE Chemical Co., Germany) (CoQ_10_: glycerol: HCO60 0.4: 0.6: 1). A 1 mg/ml stock solution was kept at −80 °C until use. Oxidation under these conditions was minimal for several months.

### Cell viability

Viability was estimated flow-cytometrically using VIA Count Millipore cytotoxicity test as previously reported (62). Briefly, the assay exploits a mixture of cell membrane permeable (red) and cell membrane impermeable (yellow) DNA-binding fluorescent probes diluted 1:10 in PBS and used to stain approximately 20000 cells immediately before reading. The analysis of the distribution allows the discrimination of the percentage of cell debris (R-/Y-), live cells (R+/Y-) and dead cells (R+/Y+).

### Total cellular ROS levels

Intracellular reactive oxygen species were quantified using the *leuco* dye, carboxy-2,7-dichlorofluorescein diacetate (carboxy-H_2_DCFDA) (Invitrogen), as previously described [62]. Briefly, statin treated adherent cells and untreated controls were washed with PBS, incubated with 10 µM carboxy-H_2_DCFDA in PBS in the dark for 30 min at 37°C. Detached cells were counter stained with Viacount reagent, and analyzed on a Guava Easycite flow cytometer (Millipore). A region representative of low levels of fluorescence was arbitrarily defined, using a gate relative to 50% of the population of untreated cells in a reference experiment (Fig. 3A). These settings were then maintained for all subsequent experiments for untreated and statin treated cells, and the relative percentage of cells for the low ROS region was calculated.

### Mitochondrial ROS production

Mitochondrial reactive oxygen species production, mainly represented by superoxide anion, was evaluated flow-cytometrically by means of the reduced nerstian probe MitoSOX™ Red (Life technologies) as previously reported [63]. Briefly, statin treated adherent cells and untreated control cells were incubated for 15 min in a solution 5 µM MitoSOX in PBS at 37°C. After trypsinization and washing cells were analyzed on a Guava Easycite flow cytometer (Millipore). In order to determine mitochondrial superoxide production, a region of fluorescence relative to high superoxide anion producing cells were defined in a reference experiment comparing untreated (negative control) and rotenone stimulated cells as positive control (rotenone is a selective mitochondrial complex I inhibitor used at 0.6 μM for 72 h). Fluorescence gates relative to high superoxide anion production were set in order to include 8% of negative control and 16% of positive controls (Fig. 3A). These settings were then maintained for all subsequent experiments.

### Permeability transition pore assessment

Mitochondrial permeability transition pore (mPTP) was assessed by evaluating the fluorescence of calcein in association with the quencher cobalt chloride (MitoProbe™ Transition Pore Assay Kit, Life technologies). Fibroblasts were washed in PBS without calcium and incubated with calcein 0.25 μM and 0.4 mM CoCl_2_ in PBS with 1.3 mM calcium for 15 min at 37°C in the dark. Trypsinized and washed cells were analyzed on a Guava easycyte flow cytometer. For data analysis, regions of fluorescence were set using as a reference control cells stained either with calcein alone (total cellular fluorescence); calcein and CoCl_2_ (mitochondrial fluorescence proportional to mitochondrial pore opening); or calcein, CoCl_2_ and calcium ionophore ionomycin (maximal quenching of mitochondrial fluorescence). The percentage of mitoprobe positive cells was therefore arbitrarily set in the untreated control population to 20%. These settings were then maintained for all subsequent experiments.

### NRF2 nuclear translocation

Simvastatin treated (20 and 40 nM) and unexposed control cells were processed according to Sunitha et al. [64]. Methanol-fixed and triton-permeabilized fibroblasts were incubated with anti-NRF2 antibody (1:400, sc-365949, Santa Cruz Biotechnology, CA, USA) overnight at 4°C (64). Cells were washed twice with PBS and then stained with FITC conjugated anti-mouse secondary antibody (1:250) for 2 h, after which the cells were stained with DAPI (300 ng/ml) for 5 min at room temperature. Nuclear translocation was assessed in fixed cells samples using Biotek Lionheart automated fluorescence microscopy system and Gene5 image analysis software. The ratio of FITC green fluorescence was calculated on 400 cells per sample and was expressed as nuclear/cytoplasm ratio through double ROI definition. Ratio values were exported to GraphPad software, plotted as box-plot and statistical differences were calculated using ANOVA and corrected for multiple comparisons using Dunnet test.

### Mitochondrial DNA content

Mitochondrial/genomic DNA ratio was used as a quantitative method to assess the cellular mitochondrial content. In particular, total DNA extracted from fibroblasts using Exgene kit (Geneall) was used to amplify and quantify reference genes present in single copies representative for nuclear DNA (fragment of actin intron sequence) and for mtDNA (COX1 region). The reported primers were used to amplify actin (forward 5'-TGACTGGCCCGCTACCTCTT-3'; reverse 5'-CGGCAGAAGAGAGAACCAGTGA-3') and COX1 (forward 5'-CTGCTATAGTGGAGGCCGGA-3'; reverse 5'-GGGTGGGAGTAGTTCCCTGC-3'), respectively. About 10 ng of DNA were amplified using 1x SYBR PCR SensiFast master mix (BIOLINE), quantification reactions were carried out using the following conditions: 95°C for 10 min followed by 95°C for 30 s and 60°C for 30 s for 40 cycles. Quantification of relative copy number differences was carried out using the delta delta Ct method.

### Cellular CoQ_10_ content

Cellular CoQ_10_ content was quantified from fibroblasts grown in 6-well plates as previously described [65]. Briefly, 50 µl cell suspension (approximately 200,000 cells) were extracted with 250 µl isopropanol. Centrifuged isopropanol extracts were injected into a high-performance liquid chromatograph (HPLC) with electro-chemical detector (ECD) by Shiseido Co. Ltd. (Tokyo, Japan) characterized by a post-separation reducing column (Shiseido CQR) capable of fully reducing eluted ubiquinone. CoQ_10_ oxidative status was expressed as percentage of ubiquinone/total CoQ_10,_ while total CoQ_10_ content in fibroblasts was expressed as µg/mg CoQ_10_/ protein.

### Mitochondrial respiration measurements

Confluent fibroblasts were grown on 6-well plates in the presence of simvastatin for 48 h. Subsequently, cells were trypsinized and seeded in XF96 well plates with 30,000 cells/well, and after attachment, treated with simvastatin for 24 h. Untreated control cells were processed in the same way.

For real-time analysis of oxygen consumption rates (OCR), fibroblasts were treated with XF Running Buffer (XF media, 5.5 mM glucose, 2 mM L-glutamax, 1 mM sodium pyruvate) according to the manufacturer’s instructions. Before analysis, the XF plate was incubated for 1 h at 37°C w/o CO_2_. OCR was expressed as pmol O_2_/min.

During respirometry, cells were sequentially treated with oligomycin (1 µM), carbonyl cyanide p-(trifluoromethoxy)-phenyl-hydrazone (FCCP; 0.6 µM), or antimycin A/rotenone (1 µM). A sample diagram of the respiratory profile is reported in Figure 6 showing oxygen consumption rates (OCR) expressed in pmol O_2_/min during all the experimental procedure in presence or in absence of simvastatin 2500 nM. After respirometry measurements, the supernatant was replaced by propidium iodide staining solution (20% (v/v) ethanol in 1.0 mg/mL propidium iodide solution in water (Life Technologies)). The plate was sealed with sealing film, protected from light using an aluminum foil cover and stored at 4°C until further use. Analysis was performed utilizing image-based fluorescence device (Spectramax Minimax 300 Imaging Cytometer, Molecular Devices) for the determination of the number of cells in each well. OCR values were normalized accordingly (pmol O_2_/min/1000 cells) (Fig. 6).

### Senescence-associated β-galactosidase activity

β-galactosidase activity in adherent statin-treated and control fibroblasts was used as a marker of cellular senescence and was quantified using senescence detection kit ab65351 (ABCAM). Briefly, samples were washed with PBS and treated with fixative solution for 10 min. After treatment, cells were washed using PBS and treated with a staining solution with substrate X-gal at 1 mg/ml. Subsequently, the plate was sealed with a plastic envelope and incubated at 37°C for one night. After incubation samples were observed with a microscope at 200x magnification. Data are expressed as % of β-galactosidase positive cells out of total cells.

### Quantitative RT-PCR for mRNA expression

Total RNA (1 μg) was reverse-transcribed with One Script Reverse Transcriptase Kit (Abm). qPCR reactions were conducted on Rotor Gene Q 5plex HRM (Qiagen) in a 10 μl total reaction volume using SYBR Green JumpStart Taq ReadyMix (S4438 - Sigma-Aldrich). GAPDH was used as a reference gene for normalization. Each reaction was run in duplicate and always included a no-template control. qRT-PCR reactions included 2 min denaturation step at 95°C for polymerase activation followed by 40 cycles of 10 sec denaturation at 95°C, 30 sec of annealing at 72°C and 30 sec extension at 60°C, during which fluorescence was measured. mRNA quantification was assessed using the 2^−ΔCt^ method.

The primers sequences (written 5’-3’) were: p16 Forward: CATAGATGCCGCGGAAGGT; GAPDH Forward: TCCTCTGACTTCAACAGCGAC, p16 Reverse: CTAAGTTTCCCGAGGTTTCTCAGA; GAPDH Reverse: TCTCTCTTCCTCTTGTGCTCTT .

### Western blot analysis

Whole-cell lysates for Western blot were obtained using RIPA Buffer (1% Triton X-100, 50 mM Tris-HCl, 150 mM NaCl, 0.1% SDS, 0.5% sodium deoxycholate, and protease inhibitor cocktail (Roche)). 20 μg of proteins were resuspended in Laemmli buffer and directly loaded onto polyacrylamide gel (15%). For Western blot analysis, the antibodies used were: p16 (1:100) (sc-377412) and anti-GAPDH (1:1000) (Santa Cruz Biotechnology, CA, USA). All primary antibodies were probed with a secondary horse radish peroxidase (HRP) conjugated antibody (Bio-Rad). Chemiluminescent assay was used for detection (Pierce, Thermo Fisher Scientific, Rockford, USA).

Levels of p16 and GAPDH were quantiﬁed by densitometry using Image J. The quantity of p16 in cells was expressed as the ratio of p16 to GAPDH.

### Statistical analysis

All experiments were performed at least in three biological replicates conducted in different experimental sessions. Data distribution of the results has been reported as box-plots and statistical significance in the differences of the mean distribution has been assessed using one-way ANOVA with post-hoc significance analysis using Dunnett test (significance level < 0.05).

## SUPPLEMENTARY MATERIAL

Supplementary Figure S1
