# Supplementary material for: Modulation of Coenzyme Q10 content and oxidative status in human dermal fibroblasts using HMG-CoA reductase inhibitor over a broad range of concentrations. From mitohormesis to mitochondrial dysfunction and accelerated aging
Source: Aging (Albany NY). 2019 May 10;11(9):2565–82. doi: 10.18632/aging.101926 (PMC6535058; doi:10.18632/aging.101926)
Supplement: Supplementary Figure S1 [file aging-11-101926-s001.pdf]

SUPPLEMENTARY MATERIAL

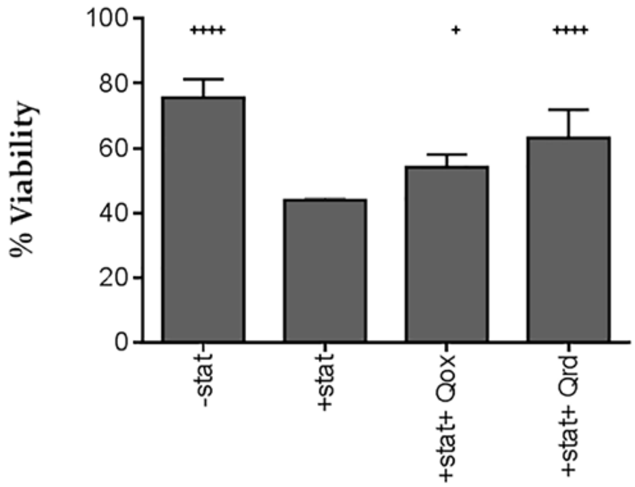

Figure S1. Rescue of simvastatin-associated cytotoxicity by ubiquinone and ubiquinol supplementation. Human dermal fibroblasts treated with simvastatin (20000 nM) alone or in presence of Coenzyme Q_{10} (ubiquinone Qox; ubiquinol Qrd 10 µg/ml) for 72 h. Data (n=4) are reported as mean and standard error of % live cells. Significance difference vs +stat $^{+}$ $p<0.05$, $^{++}$ $p<0.01$, $^{+++}$ $p<0.001$, $^{++++}$ $p<0.0001$.
